# Resistance to organic hydroperoxides requires *ohr *and *ohrR *genes in *Sinorhizobium meliloti*

**DOI:** 10.1186/1471-2180-11-100

**Published:** 2011-05-13

**Authors:** Catherine Fontenelle, Carlos Blanco, Morgane Arrieta, Virginie Dufour, Annie Trautwetter

**Affiliations:** 1 UMR CNRS 6026, DUALS. Université de Rennes I, Campus de Beaulieu, Av. du Général Leclerc, 35042 Rennes, France

## Abstract

**Background:**

*Sinorhizobium meliloti *is a symbiotic nitrogen-fixing bacterium that elicits nodules on roots of host plants *Medicago sativa*. During nodule formation bacteria have to withstand oxygen radicals produced by the plant. Resistance to H_2_O_2 _and superoxides has been extensively studied in *S. meliloti*. In contrast resistance to organic peroxides has not been investigated while *S. meliloti *genome encodes putative organic peroxidases. Organic peroxides are produced by plants and are highly toxic. The resistance to these oxygen radicals has been studied in various bacteria but never in plant nodulating bacteria.

**Results:**

In this study we report the characterisation of organic hydroperoxide resistance gene *ohr *and its regulator *ohrR *in *S. meliloti*. The inactivation of *ohr *affects resistance to cumene and ter-butyl hydroperoxides but not to hydrogen peroxide or menadione *in vitro*. The expression of *ohr *and *ohrR *genes is specifically induced by organic peroxides. OhrR binds to the intergenic region between the divergent genes *ohr *and *ohrR*. Two binding sites were characterised. Binding to the operator is prevented by OhrR oxidation that promotes OhrR dimerisation. The inactivation of *ohr *did not affect symbiosis and nitrogen fixation, suggesting that redundant enzymatic activity exists in this strain. Both *ohr *and *ohrR *are expressed in nodules suggesting that they play a role during nitrogen fixation.

**Conclusions:**

This report demonstrates the significant role Ohr and OhrR proteins play in bacterial stress resistance against organic peroxides in *S. meliloti*. The *ohr *and *ohrR *genes are expressed in nodule-inhabiting bacteroids suggesting a role during nodulation.

## Background

*Sinorhizobium meliloti *is a soil bacterium that must survive and proliferate in various adverse conditions. *S. meliloti *is also able to establish a symbiotic partnership with *Medicago sativa *leading to the formation of nodules. In nodules, the bacterium differentiates in bacteroids and fixes atmospheric nitrogen. Within the soil and during nodulation, *S. meliloti *copes with various stresses imposed by the environment [1] or by plant responses to bacterial invasion [2,3]. While nodulation is a close association between plant and *S. meliloti*, bacteria are initially recognised as intruders and induce an oxidative burst [4].

An increased production of reactive oxygen species (ROS), including superoxides, H_2_O_2 _and organic hydroperoxides is an important component of plant defences [5]. In free living bacteria oxidative stress results from aerobic metabolism [6], ROS are produced as a result of the incomplete reduction of molecular oxygen [7]. Hence, both in free living and symbiotic stages, *S. meliloti *produces enzymes to detoxify ROS. Only those that detoxify superoxide anion and H_2_O_2 _have been studied extensively Superoxides are detoxified by two superoxide dismutases [8,9], H_2_O_2 _by three catalases (KatA, KatB and KatC) [10] and a chloroperoxidase (Cpo) [11]. Little is known about resistance to organic peroxides (OHPs) in *S. meliloti*. OHPs are generated as part of the active defence response of plants [12,13]. OHPs are highly toxic. They participate in free radical reactions that generate more toxic ROS by reacting with membranes and other macromolecules [14]. Thus, detoxification of OHPs is important for bacterial survival and proliferation.

Bacteria possess two systems to protect themselves against organic peroxide toxicity. Peroxiredoxines have been shown to be the main peroxide detoxification enzymes in eukaryotes and bacteria [15,16]. Alkyl hydroperoxidase reductase (Ahp) constitutes the best characterised member of peroxiredoxin family [17,18]. This enzyme is composed of a reductase subunit and a catalytic subunit reducing organic peroxides to alcohols [18]. The second class of OHP detoxification enzymes (OsmC/Ohr family) is only found in bacteria [19]. The Ohr (Organic Hydroperoxide Resistance) protein first discovered in *Xanthomonas campestris *[20], and OsmC (Osmotically inducible protein) [21] are hydroperoxide peroxidases catalysing the reduction of hydroperoxides into their corresponding alcohols [22,23]. Both Ohr and OsmC are structurally and functionally homologous proteins. They are homodimeric with the active sites on either side of the molecule [23,24]. Their active sites contain two highly conserved cysteines which are involved in peroxide metabolism [24,25]. Despite this conservation of the proteins, OsmC and Ohr display different patterns of regulation and distinct physiological functions [23]. The expression of *ohr *is specifically induced by organic peroxides and not by ethanol and osmotic stress [19], while *osmC *is not induced by organic peroxides; instead it is induced by ethanol and osmotic stress and controlled by multiple general stress responsive regulators [15]. The inactivation of *ohr*, but not *osmC*, reduces the resistance only against organic peroxides, and not to other oxidants [20].

The expression of *ohr *is regulated by the organic peroxide-inducible transcription repressor OhrR, a member of MarR family. Structural data are available for OhrR of *Bacillus subtilis *[26] and OhrR of *X. campestris *[27]. OhR functions as a dimeric repressor that binds the *ohr *promoter region in the absence of organic peroxides. Derepression results from the oxidation of a highly conserved active site cysteine that resides near the NH_2 _terminus of the protein [28]. *B. subtilis *OhrR contains only one cysteine; its oxidation leads to the formation of a mixed disulfide with a low molecular weight thiol, a cyclic sulfenamide, or overoxidation to sulfinic or sulfonic acids [29]. In other bacteria, like *X. campestris*, OhrR contains a second cysteine located on the COOH extremity of the OhrR protein (C127 for *X. campestris*). Oxidation of the protein initiates by the formation of a sulphenic derivative of the reactive cysteine (C22) followed by the formation of a disulfide bond with C127 of the other OhrR subunit [30]. While *ohr *homologues are widely distributed in bacterial genomes [19], the role of *ohr *and *ohrR *was only studied in a few number of bacteria: *X. campestris, B. subtilis, Agrobacterium tumefasciens, Pseudomonas aeruginosa *and *Streptomyces coelicolor *[20,31-35].

In many bacteria, peroxide stress was studied only via H_2_O_2 _stress. In *S. meliloti*, H_2_O_2 _resistance has been extensively studied [8,10,11] while OHP resistance is poorly understood. This study aims at evaluating the role of *ohr *and *ohrR *genes on OHP resistance in *S. meliloti*. The analysis of the biochemical properties of *ohr *and *ohrR *mutants and the expression pattern suggests that this system should play an important role in sensing and protection of *S. meliloti *from OHPs.

## Results

### Identification of Ohr and OhrR homologues in *S. meliloti*

Blast search of *S. meliloti *genome for homologues of *X. campestris *Ohr protein revealed two paralogues, SMa2389 and SMc00040, showing 52 and 57% identity respectively with Ohr of *X. campestris*. They possess conserved active site cysteines of Ohr/OsmC proteins [19]. SMa2389 is annotated as OsmC. SMc00040 has been shown to be induced by peroxide stress [11]; it is divergently located from a gene encoding a MarR family regulator that has 49 and 45% identity with the OhrR regulatory protein of *X. campestris *and *B. subtilis *respectively. SMc01945 has been previously published as OhrR like repressor since it presents 40% identity with OhrR of *X. campestris *[11]; the adjacent gene *cpo *(SMc01944) has been shown to encode a secreted peroxidase.

Co-localisation on the genome of *ohr *and *ohrR *was found in all bacteria in which these genes were investigated [20,31,36], suggesting that SMc00040 and SMc00098 encodes respectively Ohr and OhrR proteins.

### *ohr *mutant growth is inhibited by organic peroxides

In order to investigate the role of *ohr *(SMc00040) and *ohrR *(SMc00098) in oxidative stress defence, *S. meliloti *strains with an *ohrR *deletion or carrying an insertion in *ohr *were constructed. The ability of these mutants to resist exposure to oxidants was evaluated; neither of the two had any growth defect when grown aerobically in complete medium LB or in minimal medium GAS. Moreover they possessed the same plating efficiency as wild type strain.

The influence of organic peroxides on growth of wild type, *ohr *and *ohrR *strains was analysed by adding increasing amounts of t-butyl hydroperoxide (tBOOH) and cumene hydroperoxide (CuOOH) to LB medium and determining the maximal OD_570 nm _reached by the cultures. A concentration of 2 mM tBOOH abolished the growth of *ohr *mutant while the growth yield of *ohrR *and parental strains was unaffected. Similarly 0.5 mM of CuOOH abolished growth of *ohr *strain but did not affect growth yield of *ohrR *and parental strains.

**Figure 1:**
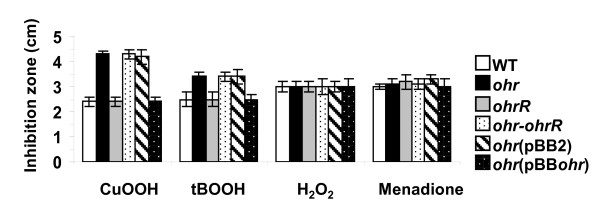
**Resistance of the *ohr *and *ohrR *mutants to ROS**. The resistance of wild type (WT), *ohr, ohrR, ohr-ohrR *mutants and *ohr *mutant complemented by plasmids pBBR1-MSC2 [*ohr *(pBB2)] and pBBohr [*ohr *(pBBohr)] was analysed by disk diffusion assay on LB plates as described in methods. The data correspond to five independent experiments; standard deviation is indicated (bars).

Disk diffusion assays were used to determine if *ohr *and *ohrR *mutations affected resistance to ROS. The *ohr *mutant was less resistant than its parental strain when challenged with organic peroxides as shown by the zones of growth inhibition: 4.1 ± 0.2 cm for CuOOH and 3.1 ± 0.1 cm for tBOOH versus to 2.3 ± 0.2 and 2.5 ± 0.3 cm for wild type strain. In contrast, *ohrR *mutation did not affect the resistance of *S. meliloti *against tBOOH and CuOOH since inhibition zones were not significantly (p value ≤ 0.01) different from those of wild type strain (Figure 1). The *ohr-ohrR *mutant behaved identically to the *ohr *mutant (Figure 1).

In other experiments, *ohr *and *ohrR *mutants were complemented by the moderate copy number plasmid pBBR1-MCS2 bearing wild type alleles of *ohr *(pBBohr) or *ohrR *(pBBohrR). The empty vector did not affect the resistance of wild type or mutants against tBOOH and CuOOH. Plasmid vector carrying *ohrR*^+ ^allele also did not affect the resistance to OHPs of these three strains. In contrast the introduction of pBBohr in *ohr *mutant dramatically improved resistance to both tBOOH and CuOOH (Figure 1). These results showed that Ohr is important in the defence against organic peroxides in *S. meliloti*.

In comparison with parental strain, *ohr *and *ohrR *mutants were not significantly affected in resistance to H_2_O_2 _and menadione; inhibition zones were nearly identical for the three strains. No alteration of this resistance was observed after complementation of the mutations with pBBohr or pBBohrR. This result agrees with the role of Ohr in other organisms and its specificity for organic peroxide resistance.

### Regulation of *ohr *and *ohrR *genes

The transcriptional activity of *ohr *and *ohrR *genes was assayed in strain R7.16 carrying *ohr::lacZ *and *ohrR::uidA *transcriptional fusions in tandem with wild type copies of *ohr *and *ohrR *genes.

The expression of these fusions was analysed in LB medium and in the minimal medium GAS. No difference was observed between both media. The expression of *ohr::lacZ *and *ohrR::uidA *was followed throughout growth till the late stationary growth phase. The expression of these two genes remained constant; no variation was observed after growth arrest. Adding NaCl to the medium during exponential growth or during stationary growth phase did not affect *ohr *or *ohrR *expression (data not shown). Such a result is expected for Ohr and clearly differentiates it from OsmC proteins that are induced by NaCl [15].

**Figure 2:**
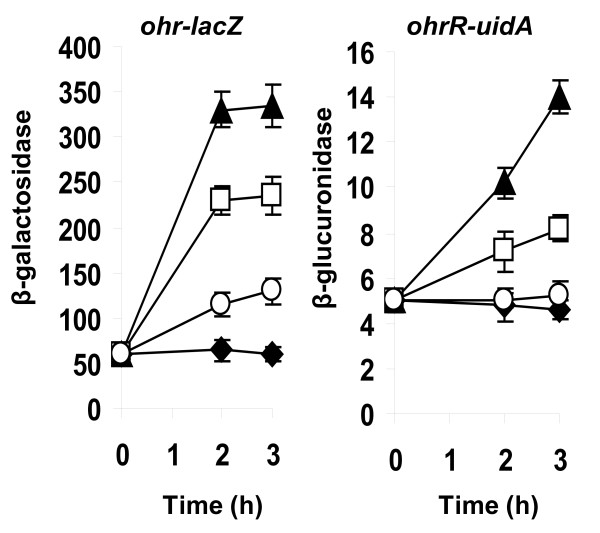
**Induction of the expression of *ohr *and *ohrR *by peroxides**. Cells were grown in LB medium to an OD_570 nm _of 0.4. *ohr::lacZ *(β-galactosidase) and *ohrR::uidA *(β-glucuronidase) expression was analysed 2 and 3 h after OHP addition. No addition (closed diamonds), 0.25 mM CuOOH (closed triangles), 1.6 mM tBOOH (open squares), 10 mM H_2_O_2 _(open circles). Enzymatic activities are expressed as nmole of substrate hydrolysed per min and per mg of protein. Results are the average of four independent experiments; the standard deviation is indicated by bars.

The influence of peroxides was analysed by introducing into the medium a concentration of peroxide that did not affect the development of exponentially growing cells. Expression of *ohr *was induced 4-fold in the presence of 1.6 mM tBOOH, a 7-fold induction was observed with 0.25 mM CuOOH. The addition of 10 mM H_2_O_2 _resulted in a 2-fold induction of *ohr *(Figure 2).

Induction of *ohrR *was also observed when cultures were exposed to tBOOH and CuOOH, induction ratios were lower than those observed for *ohr *gene. In contrast H_2_O_2 _did not affect *ohrR *expression (Figure 2).

### OhrR regulates *ohr *expression

A plasmid bearing *ohr::lacZ *transcriptional fusion (pE1541) was introduced into the *ohrR *mutant and the parental strain. The expression of the fusion was analysed in LB medium in the absence of organic peroxides and 1 h after 0.25 mM CuOOH addition. In the absence of peroxide, the expression of *ohr::lacZ *fusion was greater in the *ohrR *mutant than in the wild type strain (18.5 ± 1.3 and 9.6 ± 0.7 μmol of substrate hydrolysed min^-1 ^mg of protein^-1 ^respectively). After CuOOH addition, the expression of *ohr::lacZ *was similar in *ohrR *mutant and parental strain (16.7 ± 1.4 and 17.5 ± 1.5 μmol of substrate hydrolysed min^-1 ^mg of protein^-1 ^respectively). These results are in accordance with repression of *ohr *promoter by the OhrR regulator.

### OhrR binds to *ohr-ohrR *intergenic region

**Figure 3:**
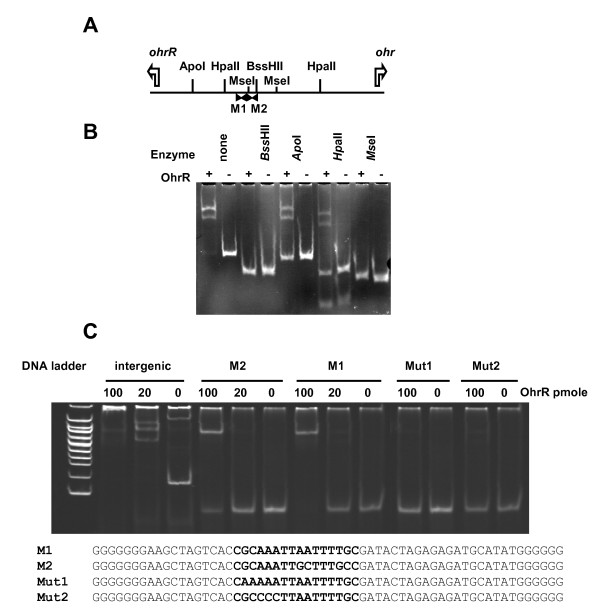
**Localisation of OhrR binding sites**. A-Restriction map of the 113 bp *ohr-ohrR *intergenic region used in gel mobility shift assay. The location of the initiator codon and translation direction of *ohr *and *ohrR *is indicated by a white arrow. The position of the two palindromic binding motifs Motif 1 (M1) and Motif 2 (M2) is indicated by black arrows. B-Gel mobility shift assay of the *ohr-ohrR *intergenic region and of its restriction fragments produced by *Bss*HII, *Apo*I, *Hpa*II or *Mse*I. DNA (20 pmoles) was incubated in the presence (+) or in the absence (-) of 20 pmoles of OhrR. C-Binding of OhrR to Motif 1 and Motif 2 sequences. Gel shift assay of the intergenic region and the 60 bp double strand sequences containing at their centre the genuine 17 nt corresponding to Motif 1 and Motif 2, or mutated Motif 1 with AA in place of GC (Mut1 fragment) and CCC in place of AAA (Mut2 fragment). DNA (20 pmoles) was incubated with the indicated amount of OhrR in the presence of 1 mM DTT.

The binding of OhrR to *ohr-ohrR *intergenic region was analysed by gel mobility shift assay. In a first attempt, a 113 bp DNA fragment encompassing the entire *ohr-ohrR *intergenic region and ended at the initiation codons of *ohr *and *ohrR*, was used as a probe (Figure 3A). Two retarded bands were observed in the presence of OhrR (Figure 3B). The intergenic region between SMb20903 and SMb20964 (this latter gene encoding the putative AhpC protein of *S. meliloti*) was used as a negative control. No specific binding of OhrR protein to this DNA fragment was observed (data not shown).

We took advantage of restriction sites located within the *ohr-ohrR *intergenic region to define further OhrR binding site. *Apo*I cleaved once this fragment giving a 17 bp and a 96 bp fragment. In the presence of OhrR protein the longer fragment produced two shifted bands (Figure 3). Two *Hpa*II sites are located within *ohr-ohrR *intergenic region; *Hpa*II cleavage produced three fragments of 26, 29 and 58 bp. In the presence of OhrR, the intensity of the 58 bp fragment decreased and two retarded bands were observed (Figure 3B). Thus OhrR binding sites are located within the 58 bp *Hpa*II fragment. None of the DNA fragments generated by *Bss*HII (54 and 59 bp) or *Mse*I (47, 50 and 16 bp, the last not detected on the gel) were shifted in the presence of OhrR (Figure 3B). The unique *Bss*HII and both *Mse*I sites are located within the 58 bp *Hpa*II region, which suggests that OhrR binding site is located within the 16 bp *Mse*I fragment or overlaps its extremities and overlaps the *Bss*HII site.

Two imperfect palindroms (Figure 3A) are located within the 58 bp *Hpa*II region. Moreover *Mse*I and *Bss*HII sites overlap these motifs. Motif 1 (GCAAATTAATTTTG) and motif 2 (GCAAATTGCTTTGC) look like the OhrR binding site GCAATT-AATTCG found in other bacteria [31,34,36,37]. Motif 1 and motif 2 are adjacent as observed for OhrR binding sites of *B. subtilis *[36], *A. tumefaciens *[31], *S. coelicolor *[34] and *X. campestris *[37].

To further analyse OhrR binding, 60 bp DNA fragments containing in their centre 17 nt corresponding either to motif 1 or motif 2 were synthesised. The OhrR protein was found to bind to both fragments. Mutations were introduced in motif 1 to confirm the importance of this sequence. The modification of GC to AA or AAA to CCC in one half of the palindrome abolishes the binding of OhrR to the DNA fragments (Figure3C).

### Modulation of OhrR activity by oxidation

*S. meliloti *OhrR protein contains two cysteine residues conserved at the same position than in OhrR of *X. Campestris*, allowing the possibility to form inter-subunit disulfide bonds upon oxidation.

**Figure 4:**
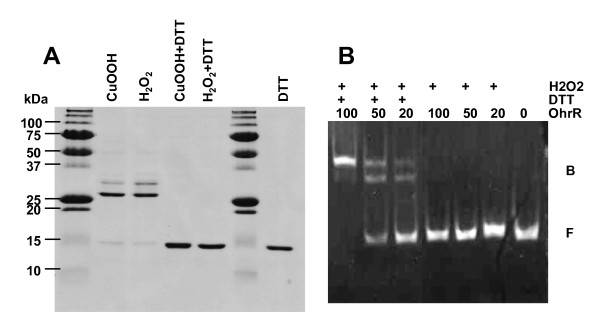
**Oxidation promotes OhrR dimerisation and inactivation**. (A) OhrR purified protein (20 nmoles) was incubated for 15 min with CuOOH (0.55 nM ) or H_2_O_2 _(0.5 nM ) and then, when indicated, added with 0.5 mM DTT and incubated for another 15 min. (B) The DNA fragment (20 pmoles) corresponding to *ohr-ohrR *intergenic region was incubated with purified OhrR protein (20, 50 or 100 pmoles) in the presence of 0.5 nM H_2_O_2 _and in the absence or in the presence of 0.5 mM DTT.

Purified OhrR was treated with CuOOH, H_2_O_2 _or DTT and the products were analysed by non reducing SDS-PAGE (Figure 4A). In the presence of DTT, *S. meliloti *OhrR protein migrated as a band of an apparent MW of 15 kDa (the calculated molecular mass being 17.5 kDa). Incubation of the protein with CuOOH or H_2_O_2 _led to the formation of a band of 30 kDa corresponding to the dimeric form. The formation of the dimer was reversed by an excess of DTT. Thus, as observed in *X. campestris *[30], the oxidation of OhrR induces a reversible bonding between the two subunits of the protein (Figure 4A).

Binding of OhrR to *ohr-ohrR *intergenic region was suppressed when 10 mM H_2_O_2 _was added to the binding mixture. Binding was recovered after addition of an excess of DTT. Thus only the reduced form of OhrR was able to bind DNA (Figure 4B).

### *ohr *strain forms fix^+ ^nodules in alfalfa

The sensitivity of *S. meliloti ohr *mutants to OHPs is potentially relevant to symbiosis since legume root cells respond to rhizobial infection with an enhanced production of ROS [4,38]. To test the effect of *ohr *mutation on nodulation and nitrogen fixation, one week old seedlings of *Medicago sativa *were inoculated with either the *S. meliloti ohr *mutant or the parental strain. Plants were grown in nitrogen-deprived medium. Five weeks after the inoculation, plants were visually screened for nodulation by observing the root system. A highly efficient nodulation was observed on plants inoculated with either *ohr *or parental strains. No significant difference between dry weights of plant shoots was observed. The inoculated plants had green leaves and comparable number of nodules, whereas the non-inoculated control plants were smaller, with yellow leaves and significantly lower dry weight. Nodules from plants inoculated with the *ohr *mutant were crushed and the bacteria recovered by plating on MSY plates before assayed for gentamycine resistance and OHP sensitivity. All the randomly selected colonies that were analysed were able to grow on gentamycine-containing plates and behaved like the original *ohr *mutant. Thus N_2_-fixing nodules formed on alfalfa were due to infection by the *ohr *mutant and not by revertants.

**Figure 5:**
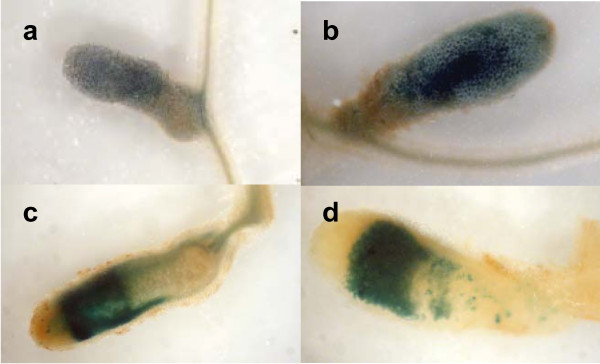
**Expression of *ohr *and *ohrR *in planta**. Nodules were fixed and stained with 5-bromo-4-chloro-3-indolyl-beta-D-galactopyranoside (β-galactosidase detection) (a,c) or 5-bromo-4-chloro-3-indolyl-beta-D-glucuronate (β-glucuronidase detection) (b, d) and visualised by light microscopy. (a, b) whole nodules, (c, d) thin sections of stained nodules. The images are representative of 30 nodules analysed.

In order to analyse *oh*r and *ohrR *expression *in planta*, β-galactosidase and β-glucuronidase activity were visualised by light microscopy on entire and sections of nodules from R7.16 (*ohr-lacZ, ohrR-uidA, ohr^+^, ohrR*^+^) infected plants (Figure 5). No staining was observed in root hairs or infection threads. Nodule staining co localises with pink coloration of leghemoglobin, corresponding to nitrogen fixation zone (data not shown). Thus, in spite of the absence of a nodulation defect of *ohr *strain, both *ohr *and *ohrR *genes were expressed during nodulation. This result is in accordance with the detection of Ohr protein in nodules in proteomic studies [39].

## Discussion

In this study, we analysed the role of *ohr *and *ohrR *genes in *S. meliloti*. As many bacteria, *S. meliloti *must survive oxidative stress generated by the environment or during symbiosis. ROS attack of cellular membranes generates a cascade of radicals leading to the formation of OHPs [7]. Moreover, OHPs are produced by plants as part of the defence response against bacteria [12,13]. Organic peroxides are potent effectors of *ohr *system in bacteria [40].

Ohr is not essential for nodulation. Bacteria containing *ohr *mutations formed effective nodules, suggesting that *S. meliloti *does not undergo OHP stress during nodulation or that other enzymes detoxify OHP like AhpC (a putative ahpC gene: SMb20964 was annotated) as described in *X. campestris *[41]. The redundancy of enzymatic activities was also described for catalases in *S. meliloti*; only strains affected at least for two catalases are compromised in symbiosis [10]. Both *ohr *and *ohrR *are specifically induced by OHPs and are expressed in nodules but no OHP detection was reported, so we could not exclude the existence of other compounds inducing *ohr *and *ohrR*.

Like in many bacteria, *ohr *is located at the immediate vicinity of its regulator: *ohrR *(SMc00098). This ORF encodes a regulatory protein of the MarR family as all known OhrR regulators. The regulator OhrR is a dimeric regulatory protein that senses organic peroxides. Two families of OhrR proteins exist; they are exemplified by OhrR of *B. subtilis *and OhrR of *X. campestris*. These two proteins share 40% amino acid identity and are structurally similar [26,27]. Nevertheless, they differ in their peroxide sensing mechanisms. The *B. subtilis *OhrR protein family contains only one cysteine residue. Depending on the oxidant, OhrR gives reversible oxidised derivatives or functions as a sacrificial regulator [42]. The *X. campestris *OhrR possesses another important cysteine (C127). The initially oxidized cysteine (C22) forms intersubunits disulfide bonds with the residue C127 on the second subunit of the dimer, leading to reversible inactivation of the protein [30]. The introduction of a second cysteine into *B. subtilis *OhrR (position 120 to 124) allows *B. subtilis *OhrR to function as *X. campestris *OhrR, protecting the protein against irreversible oxidation in presence of strong oxidants [43]. Comparison of *S. meliloti *OhrR protein with that of *B. subtilis *and *X. campestris *shows that *S. meliloti *protein keeps similar amino acid identity with both proteins (45 and 49% respectively). *S. meliloti *possesses two cysteines at the same position than OhrR of *X. Campestris*. We observed that protein oxidation led to the formation of a dimer and loss of DNA binding, these phenomena are reversed by DTT in vitro. Thus *S. meliloti *OhrR oxidation mechanism is similar to that described for OhrR of *X. campestris*.

The expression of *ohr *and *ohrR *was assayed at the transcriptional level. Their expression was constant throughout growth and no induction during stationary growth phase was observed. Similarly, osmotic stress did not induce *ohr *or *ohrR *expression. These observations match with the expression of these genes in *X. campestris, A. tumefasciens, B. subtilis, P. aeruginosa *and *S. coelicolor *[20,31,32,34,44]. As previously observed in these bacteria, *ohr *and *ohrR *genes of *S. meliloti *were induced by tBOOH and CuOOH. H_2_O_2 _was a poor inducer of *ohr *gene in *S. meliloti*. Induction of *ohr *by H_2_O_2 _in other bacteria is contradictory. Western analysis and gene fusion assays showed that *ohr *is not induced by H_2_O_2 _in *A. tumefasciens, B. subtilis, P. aeruginosa *and *S. coelicolor *[31,33,34,36] and only *X. campestris ohr *is slightly induced by H_2_O_2 _[20]. Transcriptomic studies of H_2_O_2 _stress response in *B. subtilis *[32] and *P. aeruginosa *[44] showed in contrast an *ohr *induction.

Induction of *ohr *requires the oxidation of OhrR. We observed that *S. meliloti *OhrR is oxidized by H_2_O_2 _in vitro and did not bind to the operator when incubated with H_2_O_2_. Nevertheless, H_2_O_2 _is a poor inducer of *ohr in vivo *and is not an inducer of *ohrR *expression. H_2_O_2 _also causes a loss of *B. subtilis *OhrR binding to *ohrA *promoter *in vitro *while *in vivo *derepression of *ohrA *upon exposure to H_2_O_2 _was not observed [28,36]. The role of H_2_O_2 _in alfalfa during symbiosis is not restricted to plant defence against bacteria. It is also important for symbiotic process [45]. H_2_O_2 _is necessary for cell wall formation and infection thread rigidity [4]. Production of H_2_O_2 _was detected in root hairs, infection threads, infection and senescence zones but not in fixing zone [46]. The expression of *ohr *and *ohrR *was detected only in nitrogen fixing zone, thus they are not expressed constitutively and they are not induced by H_2_O_2 _*in planta*. These data suggest that organic peroxides are produced in nodules so that Ohr protein plays a role during nitrogen fixation.

## Conclusions

Resistance to organic hydroperoxides has not been previously analysed in *S. meliloti*. We have demonstrated that Ohr protein is essential for *S. meliloti *to survive organic peroxide stress. The expression of *ohr *and *ohrR *genes in nodules suggests that the Ohr protein participates in organic peroxides detoxification within the nodule.

## Methods

### Bacterial strains, plasmids, and culture conditions

The bacterial strains used in this study are detailed in Table 1. *S. meliloti *strains were grown aerobically at 30°C in the complex medium LB [47] to an optical density at 570 nm (OD_570_) of 1.5 to 1.8; they were then inoculated in minimal galactose aspartate salts medium (GAS) [48] or LB medium at an OD_570 nm _of 0.1. *E. coli *strains were grown aerobically in LB medium at 37°C. For the selection of *E. coli *strains, ampicillin was added at 50 or 100 μg ml^-1^, tetracycline at 10 μg/ml, chloramphenicol at 25 μg/ml, and neomycin or kanamycin at 50 μg/ml. For the selection of *S. meliloti *strains, streptomycin was used at 100 μg/ml, tetracycline at 5 μg/ml, and neomycin at 25 μg/ml.

**Table 1:** Bacterial strains and plasmids

Strains	genotype	origin
*E. coli*		
DH5α	*endA-1 hsdR-17 supE-44 thi-1 recA-1 gyrA relA-1 Δ(lacZYA-argG)U169 deoR*	[57]
MT616	MM294 pRK600 Cm^R^	[58]
BL21(DE3)	F^- ^*dcm ompT hsd gal*/λ(DE3)	[59]
*Sinorhizobium meliloti*		
Rm1021	SU47 Sm^R^	[60]
R6.48	Rm1021*,ohrR::*Gm^R^	This study
R7.15	Rm1021*,ΔohrR ohr::*Gm^R^	This study
R7.16	Rm1021*,ohr^+ ^ohrR^+ ^ohr::lacZ ohrR::uidA*	This study
R8.39	Rm1021*,ohr::*Gm^R^	This study
Plasmids		
pGEMT	pUC derivative cloning vector, Amp^R^	Promega
pGEMTeasy	pUC derivative cloning vector, Amp^R^	Promega
pET22b+	expression vector, Amp^R^	Novagen
pK18mob*sacB*	mobilisable pUC derivative, *sacB *Neo^R^	[51]
pBBR1-MCS2	broad host range replicating mobilisable vector, Neo^R^	[61]
pBBR1-MCS5	broad host range replicating mobilisable vector, Gm^R^	[61]
pTH1505	Gm^R^, *gfp, lacZ, uidA, rfp *fusion vector	[54]
p34SGm	ori ColEI Amp^R ^Gm^R^cassette	[52]
pD3001	pK18mob*sacB (Xba*I*-Pst*I*)/ohrR *downstream region (*Xba*I*-Nsi*I)	this study
pD3083	pGEMTeasy/ *ohrR *upstream region	This study
pD4116	pK18mob*sacB *Δ*ohrR*	This study
pD4244	pK18mob*sacB *Δ*ohrR::*Gm^R^	This study
pD5333	pK18mob*sacB*ΔohrR *ohr::*Gm^R^	This study
pD5455	pTH1505 ohr::*lacZ, ohrR::uidA*	This study
pD8657	pK18mob*sacB ohr::*Gm^R^	This study
pBBohr	pBBRI-MCS2 *ohr^+^*	This study
pBBohrR	pBBRI-MCS2 *ohrR^+^*	This study
pE1541	pBBRI-MCS2 *ohr::lacZ*	This study
pETohrR	pET22b+ *ohr*^+^	This study

### DNA manipulations and mutant constructions

Standard protocols were used for DNA manipulations [49].

### β-glucuronidase and β-galactosidase assays

β-glucuronidase and β-galactosidase assays were carried out as described [47,50]. Specific activities are expressed as nanomoles of ortho-nitrophenol liberated per minute per milligram of protein. Protein concentration was determined by the method of Bradford with bovine serum albumin as a standard. Results are the mean of at least three independent experiments, and the standard deviation was less than 10%.

### Disk diffusion assay

Cells were grown in LB medium to an OD_570nm _of 0.4. 0.5 ml of cell suspension were mixed with 3 ml of soft agar (0.4%) and poured onto LB agar plates (20 ml). 10 μl of 0.1 M cumene hydroperoxyde (CuOOH), 0.5 M t-butyl hydroperoxide (tBOOH), 10 M H_2_O_2 _or 50 mM menadione were loaded on 8 mm paper disks placed on top agar. Plates were incubated for 24 h at 30°C and the clear zone was measured. CuOOH, tBOOH and menadione solutions were made in 95% ethanol. 10 μl of ethanol produced no growth inhibition in this assay.

### Construction of a ΔohrR strain

A 2,152 bp DNA fragment corresponding to the upstream region of *ohrR *was amplified on *S. meliloti *chromosomal DNA using the primers (GATCGGCCTCGACCCATACG) and (CCTCGTCTAGATGTCATTGTCG; introduces an *Xba*I restriction site in place of *ohrR *ATG initiation codon) and cloned in pGEMTeasy vector (Promega, La Jolla, CA) giving pD3083. A 1,468 bp DNA fragment corresponding to the downstream region of *ohrR *was amplified using the primers (AGCTCTAGAGCACCTGCAG; introduces an *Xba*I restriction site in place of *ohrR *stop codon) and (CAGCGCGTGTGGCGGCG). This amplicon was digested with *Xba*I and *Nsi*I (genuine site) and cloned into pK18mob*sacB *vector [51] between the *Xba*I and *Pst*I sites, giving pD3001. pD3083 and pD3001 were linearised with *Xba*I and ligated in order to assemble *ohrR*-upstream and -downstream sequences. Then pGEMTeasy vector was deleted through an *Eco*RI digest, giving pD4116, and the GmR cassette of p34SGm [52] was inserted into the *Xba*I site, giving pD4244. This final construction carries the *ohr-ohrR *region where the *ohrR *open reading frame is replaced by a GmR cassette; it was introduced into *S. meliloti *Rm1021 strain by triparental mating and recombinants were selected for on MSY medium containing gentamycin and sucrose. Double crossing over recombinants were identified as neomycin sensitive strains and confirmed by PCR. The mutation was transduced into *S. meliloti *Rm1021 strain using ΦM12 [53], yielding R6.48.

### Inactivation of *ohr*

A 4 kb chromosomal DNA fragment containing *ohr *and *ohrR *genes was amplified by PCR using the primers (GATCGGCCTCGACCCATACG) and (CAGCGCGTGTGGCGGCG) and cloned into pGEMTeasy vector. The insert was recovered with *Eco*RI and transferred to the same site on pK18mob*sacB *vector. The *ohr *open reading frame was then inactivated by introducing into the unique *Not*I site the GmR cassette from pBBR1-MCS5 digested with *Not*I. The resulting plasmid pD8657 was introduced into Rm1021 strain and double crossing events were selected as before and confirmed by PCR. The mutation was transduced into Rm1021 strain using ΦM12, yielding R8.39.

### Construction of an *ohr::*Gm^R^*, ΔohrR *strain

pD4116 carries the entire *ohr *sequence and a deletion of *ohrR*. The *ohr *gene was disrupted by introducing in its unique *Not*I site the GmR resistance cassette from pBBR1-MCS5 recovered through a *Not*I digest. The resulting pD5333was conjugated into Rm1021 strain and double crossing overs were selected as previously described and confirmed by PCR. Transduction of the mutations into Rm1021strain yielded R7.15.

### Construction of *ohr::lacZ, ohrR::uidA *into a wild type genetic background

The 4 kb chromosomal fragment amplified for *ohr *inactivation contains two *Sal*I sites near the 3'end of *ohr *and *ohrR *genes respectively. It was cleaved with *Sal*I and the 980 bp DNA fragment containing the 5' regions of *ohr *and *ohrR *was introduced into the *Xho*I site of pTH1705 vector (not replicative in *S. meliloti*) [54]. In the resulting pD5455 plasmid two transcriptional fusions are generated: *ohr::lacZ *and *ohrR::uidA*. pD5455 was introduced into Rm1021 strain by triparental mating. Single crossing over events were selected as GmR strains. Recombination was confirmed by PCR. The transduction of the mutation into Rm1021 strain yielded R7.16.

### Analysis of *ohr *regulation by OhrR

*ohr::lacZ *region was released from pD5455 using *Xba*I and *Sph*I and introduced between the corresponding sites of pBBR1-MCS2 vector (replicative in *S. meliloti*), yielding pE1541. This plasmid was introduced by triparental mating into the wild type strain and *ohrR *mutant (R6.48) and β-galactosidase activities were assayed in both strains.

### Complementation plasmids

The open reading frames of *ohr *and *ohrR *were amplified using the primers (GATCGGCCTCGACCCATACG) and (CCTCGTCTAGATGTCATTGTCG) for *ohr *and (CGTCGATAAAGAAGCCTGTG) and (CAGCGCGTGTGGCGGCG) for *ohrR*. The amplicons were cloned into pGEMTeasy, released by *Eco*RI cleavage and introduced into the same site in pBBR1-MCS2 vector. The correct orientation allowing the expression of these genes under the control of *lac *promoter was selected. The corresponding plasmids pBB*ohr *and pBB*ohrR *were introduced into Rm1021 strain and the various mutants by triparental mating.

### Purification of OhrR protein

The *ohrR *open reading frame was amplified by PCR using the primers (CGACAATGACATATGACGAGG) and (AGCTCTCGAGTCGACTACCG) and cloned in pGEMT. The insert was released as an *Nde*I-*Xho*I DNA fragment and introduced into the expression vector pET22b+ (Novagen) giving pET*ohrR *where the *ohrR *ORF is fused to a 6his-tag at its 3' extremity. BL21(DE3) cells harbouring pET*ohrR *were cultured in LB medium at 37°C until OD_570 nm _of 0.8; isopropyl-β-D-galactopyranoside was then added to a final concentration of 1 mM. The culture was grown for an additional 4 h, and cells were harvested by centrifugation (5,000 × g, 10 min, 4°C). Bacterial cells were washed in TE (10 mM Tris pH 6.8, 1 mM EDTA) and resuspended in the same buffer with 1 mM phenylmethylsulfonyl fluoride. Cells were disrupted by three passages through a French press (1,200 PSI), and cell debris were removed by centrifugation at 4°C, 12,000 × *g *for 30 min. Proteins were loaded on a heparin column (GE heath care), followed by a wash (10 column volumes) with buffer A (25 mM Tris-HCl pH8, 25 mM NaCl, 2 mM EDTA, 1 mM DTT). Elution was performed with the same buffer containing 0.5 M NaCl. The eluted fractions were analysed by SDS-PAGE, and those containing OhrR were pooled and dialysed against buffer A.

### Gel mobility shift

The intergenic region between *ohr *and *ohrR *was amplified by PCR using the primers (ATGATGTCATTGTCGCAAATTC) and (CATGACAGTCTCCTTCCTTGTG) as a 113 bp DNA fragment. Complementary oligonucleotides (Figure 4) were also used in gel mobility assay; they were annealed in 50 mM Tris-HCl pH8, 0.25 M NaCl, 1 mM EDTA. DNA probes (20 pmoles) were incubated with OhrR protein (0 to 100 pmoles) in 20 μl binding buffer (20 mM Tris-HCl (pH 8.0), 50 mM KCl, 1 mM EDTA, 50 μM bovine serum albumin) at room temperature for 10 min. Binding mixture was run on 6% polyacrylamide gel in Tris-borate buffer. Gels were stained with SYBR gold (Molecular Probes) in 10 mM TBE pH 8.0 buffer and revealed with a transilluminator at 312 nm. To oxidize OhrR, organic peroxides were added to the binding buffer; reduction of the protein was performed with DTT.

### Plant assays

*Medicago sativa *L. var. Europe (alfalfa) was used as host plant for testing nodulation of *S. meliloti *strains according to [55]. Surface-sterilized germinating seedlings were grown in test tubes on nitrogen-free medium. One week old plants were inoculated with 10^9 ^cells of wild type and *ohr *mutant of *S. meliloti*. Plants were analysed after 5 to 9 weeks of growth.

### β-galactosidase and β-glucuronidase detection in plants

Nodules were fixed and stained as previously described [56] and observed by light microscopy.

### Authors' contributions

CB performed mutants' construction, CF, MA and VD carried out experiments concerning their phenotype characterization. AT performed gel shift experiments. AT and CB discussed the results and elaborated the final version of manuscript. All authors read and approved the final version of the manuscript.
